# Esta é uma Relação Causal? Randomização Mendeliana como um Método Estatístico para Desvendar Conexões

**DOI:** 10.36660/abc.20240606

**Published:** 2024-10-24

**Authors:** Lucas Vieira Lacerda Pires

**Affiliations:** 1 Hospital das Clínicas da Faculdade de Medicina da Universidade de São Paulo Instituto do Coração São Paulo SP Brasil Instituto do Coração do Hospital das Clínicas da Faculdade de Medicina da Universidade de São Paulo, São Paulo, SP – Brasil

**Keywords:** Análise da Randomização Mendeliana, Doenças Cardiovasculares, Comportamento Sedentário

A Randomização Mendeliana (RM) é uma ferramenta estatística poderosa para inferir uma relação causal entre a presença de variantes genéticas e diferentes características e fenótipos.^1,2^ É particularmente útil ao tentar elucidar relações causais entre exposições e desfechos com base em dados observacionais. Este método se baseia no princípio da aleatoriedade de como as variantes genéticas podem se segregar durante o processo de meiose, o princípio mendeliano de sortimento independente.^2^

Desde as últimas décadas, à medida que nosso conhecimento sobre a arquitetura genética dos seres humanos foi aprimorado, o desafio é entender como variantes genéticas podem contribuir para o desenvolvimento de fenótipos e características. Aprendemos com essa compreensão crescente que, à medida que a complexidade genética de uma característica específica aumenta, ela pode ser menos hereditária, e nosso poder preditivo se reduz.^3^

Em resposta a esse efeito, várias técnicas foram desenvolvidas, como a RM. Esta técnica permite mitigar a interferência de fatores de confusão e vieses comumente apresentados por estudos observacionais.^1,2^ De fato, a avaliação adequada de todas as suposições de RM pode garantir a validade de inferências causais causadas por esse tipo de avaliação. Nos últimos anos, estudos sobre RM foram publicados em diversas áreas de estudos médicos além do campo da genética médica e genômica (como cardiologia, nefrologia e hepatologia).^1,4,5^

**Figura 1:**
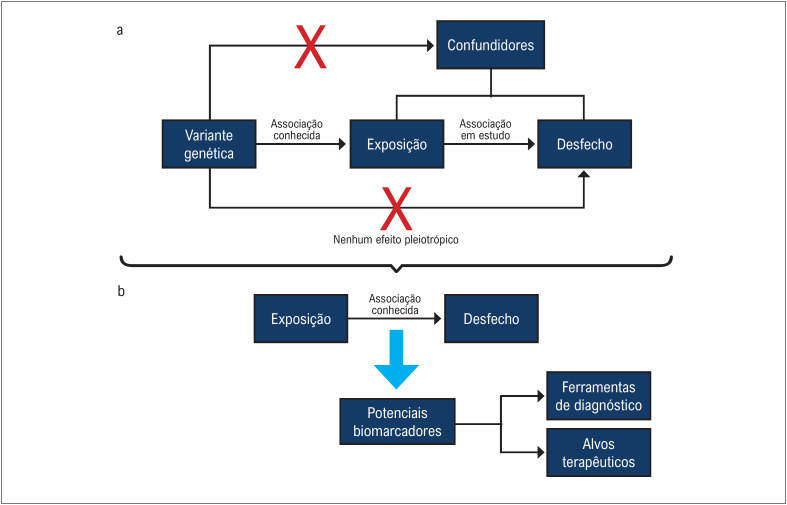
Arquitetura do fluxo de trabalho de um estudo de RM. (a) Refere-se às suposições que devem ser respeitadas para garantir sua validade para inferir uma relação causal. (b) Indica os resultados de uma RM bem projetada e os insights potenciais que podem advir da associação exposição-desfecho.

Três pilares principais compõem as suposições da RM em relação a uma variante genética conhecida que pode ser aplicada a esse tipo de estudo: a variante genética conhecida está associada à exposição de interesse; a variante genética não está associada a nenhum fator de confusão da relação exposição-desfecho; e a variante genética afeta o desfecho exclusivamente por meio da exposição, excluindo qualquer possibilidade de pleiotropia.^2,5^ A Figura 1a resume as principais premissas de um estudo de RM.

Além disso, um estudo de RM também pode inferir e possivelmente identificar potenciais biomarcadores para doenças ao alavancar variantes genéticas como variáveis instrumentais. Ao identificar variantes genéticas que influenciam eventos de exposição e subsequentemente avaliar seu impacto nos desfechos, a RM pode destacar adequadamente biomarcadores que podem ser usados posteriormente para desenvolver ferramentas de diagnóstico e possíveis alvos terapêuticos.^6-8^ A Figura 1b resume os ganhos potenciais que podem advir de um estudo de RM.

Nesse contexto, o tempo de visualização da televisão, uma medida específica do comportamento sedentário que já está associado ao aumento do risco de doenças cardiovasculares, ao risco cardiometabólico e ao aumento da mortalidade em geral,^9,10^ a RM surge como uma abordagem interessante não apenas para reforçar essa conexão, mas também para elucidar mecanismos potenciais e vias biológicas que podem estar envolvidas.^11^

Neste estudo, os autores usaram a RM para identificar não apenas uma associação entre o tempo elevado de visualização de televisão e várias doenças cardiometabólicas — consistente com a literatura existente — mas também para descobrir marcadores inflamatórios e metabólicos importantes potencialmente envolvidos nessa relação. Esses marcadores incluem níveis aumentados de interleucinas, proteína C-reativa, leptina, tecido adiposo visceral e subcutâneo, bem como índice de massa corporal, circunferência da cintura e níveis de triglicerídeos elevados. Essas descobertas sugerem mecanismos potenciais que podem ser explorados em pesquisas futuras para melhorar o diagnóstico e o tratamento ou para propor novas estratégias para o gerenciamento de pacientes com doenças cardiovasculares.

De fato, a RM pode ser um método poderoso; no entanto, algumas limitações devem ser destacadas: o principal fator que pode reduzir o poder preditivo da RM é um viés populacional. Como a maior parte da associação entre variantes genéticas e características genéticas é baseada em estudos de ancestralidade europeia, às vezes a associação não pode ser explorada para populações não europeias, especialmente naquelas com altas taxas de endogamia ou com origens genéticas misturadas, como os brasileiros. Mais estudos genéticos populacionais são necessários para estratificar e entender melhor o papel genético no desenvolvimento de características. Além disso, é importante notar que a RM é uma ferramenta poderosa para inferir associações e permite apenas a geração de hipóteses sobre potenciais biomarcadores e mecanismos; novos estudos devem surgir para certificar essas hipóteses.^12,13^
