# Supplementary material for: Esta é uma Relação Causal? Randomização Mendeliana como um Método Estatístico para Desvendar Conexões
Source: Arq Bras Cardiol. 2024 Oct 24;121(10):e20240606. [Article in Portuguese] doi: 10.36660/abc.20240606 (PMC11634222; doi:10.36660/abc.20240606)
Supplement: Translation

# Translation: Is This a Causal Relationship? Mendelian Randomization as a Statistical Method for Unraveling Connections

**DOI:** 10.36660/abc.20240606i

**Authors:** Lucas Vieira Lacerda Pires

**Affiliations:** 1 São Paulo SP Brazil Instituto do Coração do Hospital das Clínicas da Faculdade de Medicina da Universidade de São Paulo, São Paulo, SP – Brazil

**Keywords:** Mendelian Randomization Analysis, Cardiovascular Diseases, Sedentary Behavior

Mendelian Randomization (MR) is a powerful statistical tool to infer a causal relationship between the presence of genetic variants and different traits and phenotypes.^1,2^ It is particularly useful when trying to elucidate causal relationships between exposures and outcomes based on observational data. This method bases itself on the principle of randomness of how genetic variants might segregate during the process of meiosis, the Mendelian principle of independent assortment.^2^

Since past decades, as our knowledge about the genetic architecture of human beings has been enhanced, the challenge is to understand how genetic variants may contribute to the development of phenotypes and traits. We have learned from this growing comprehension that as the genetic complexity of a specific trait rises, it may be less inheritable, and our predictive power reduces.^3^

In response to that effect, several techniques have been developed, such as MR. This technique allows to mitigate the interference of confounder factors and biases commonly presented by observational studies.^1,2^ Indeed, the proper evaluation of all MR assumptions may ensure the validity of causal inferences caused by this kind of evaluation. In the past years, studies regarding MR have been published in several areas of medical studies besides the field of medical genetics and genomics (such as cardiology, nephrology, and hepatology).^1,4,5^

Three main pillars compound the assumptions of MR regarding a known genetic variant that can be applied to that kind of study: the known genetic variant is associated with the exposure of interest; the genetic variant is not associated with any confounder factor of the relationship exposure-outcome; and the genetic variant affects the outcome exclusively through the exposure, excluding any possibility of pleiotropy.^2,5^ Figure 1a resumes the main assumptions of an MR study.

**Figure 1:** Workflow architecture of an MR study. (a) Refers to the assumptions that must be respected to guarantee its validity to infer a causal relationship. (b) Indicates the results of a well-designed MR and potential insights that can come from the exposure-outcome association.

Moreover, an MR study can also infer and possibly identify potential biomarkers for diseases by leveraging genetic variants as instrumental variables. By identifying genetic variants that influence exposure events and subsequently assessing their impact on outcomes, MR can properly highlight biomarkers that can later be used to develop diagnostic tools and possible therapeutic targets.^6-8^ Figure 1b summarizes potential gains that can come from an MR study.

In that context, television viewing time, a particular measure of sedentary behavior which is already associated with increased risk of cardiovascular diseases, cardiometabolic risk, and increased mortality at all,^9,10^ MR emerges as an interesting approach not just to reinforce this connection but also to elucidate potential mechanisms and biological pathways that may be involved in.^11^

In this study, the authors used MR to identify not only an association between elevated television viewing time and several cardiometabolic diseases—consistent with existing literature—but also to uncover key inflammatory and metabolic markers potentially involved in this relationship. These markers include increased levels of interleukins, C-reactive protein, leptin, visceral and subcutaneous adipose tissue, as well as elevated body mass index, waist circumference, and triglyceride levels. These findings suggest potential mechanisms that could be explored in future research to improve diagnosis and treatment or to propose novel strategies for managing patients with cardiovascular disease.

Indeed, MR may be a powerful method; however, some limitations must be highlighted: the main factor that may reduce MR predictive power is a population bias. As most of the association between genetic variants and genetic traits is based on studies of European ancestry, sometimes the association cannot be exploited to non-European populations, especially in those with high inbreeding rates or with admixed genetic backgrounds such as Brazilians. Further populational genetic studies are needed to stratify and better understand the genetic role in developing traits. Also, it is important to notice that MR is a powerful tool to infer associations and only allows the generation of hypotheses about potential biomarkers and mechanisms; new studies must come over to certify these hypotheses.^12,13^
